# Effect of Pre-Blended Phosphates on the Freezing Quality Characteristics of Ground Woody Breast Meat Compared to Normal Meat

**DOI:** 10.3390/ani10101880

**Published:** 2020-10-15

**Authors:** Laura J. Garner, Lasheda Brooks, Lindsey F. Spencer, John Rehm, Jasmine Kataria, Amit Morey

**Affiliations:** 1 Department of Poultry Science, Auburn University, 260 Lem Morrison Drive, Auburn, AL 36849, USA; bauerlj@auburn.edu (L.J.G.); lab0086@auburn.edu (L.B.); lfs0010@auburn.edu (L.F.S.); jgr0012@auburn.edu (J.R.); 2 Department of Food Science, University of Georgia, 110 Cedar St., Athens, GA 30602, USA; jasmine.k@uga.edu

**Keywords:** Woody Breast, Ground Poultry Meat, Cryoprotectant, Sodium Tripolyphosphate, Frozen Storage

## Abstract

**Simple Summary:**

Woody breast (WB) myopathy is a major meat quality issue facing the global poultry industry. The WB affected meat can be ground, frozen, and utilized for further processed poultry products as needed. Freezing and thawing of WB meat may affect the functional properties of myofibrillar proteins such as lower water holding capacity and gelation. We found that pre-blended phosphate can increase the functionality of freshly ground WB meat and maintain it after freezing. We have demonstrated that the addition of phosphates can help the utilization of ground woody breast meat even after frozen storage.

**Abstract:**

Woody breast (WB) myopathy affected meat has a tough texture, higher cook loss, and decreased water holding capacity (WHC), and thus lower consumer acceptability. The WB meat can be ground and further converted into further processed products or frozen, stored, and shipped to further processors. Freezing and thawing of ground WB meat may further affect the quality of WB meat products. Hence, research is required to determine the effect of pre-blended phosphates on the quality of ground WB meat as well as its cryoprotective effect during frozen storage. The objective of this experiment was to investigate the effect of pre-blended phosphate levels on meat quality in WB and normal breast (NB) fillets before and after freezing. NB fillets and severely affected WB fillets were procured from a local commercial processor. The meat was separated into various treatment groups according to the sodium tripolyphosphate (STPP) inclusion levels (0, 0.25, and 0.5% w/w). The meat was ground with respective phosphate treatments and subdivided into vacuum-sealed bags (*n* = 240; 1 kg each). Half of the bags (*n* = 120) from each treatment were taken for meat quality analysis, while the other bags were placed in a freezer (−18 °C) for 6 days. Fresh samples were analyzed within 6–8 h while the frozen samples were thawed for 18 h at 4°C prior to analysis. Samples (*n* = 10) were analyzed for gel strength, pH, color (L* a* b*), proximate composition, and randomly selected samples (*n* = 5) were analyzed for aerobic plate count (APC). Experiments were repeated in two separate replications and the data was analyzed using the Proc Glimmix model procedure in SAS (v. 9.4) (Cary, NC, USA) with LSMeans Separation at *p* ≤ 0.05. The gel strength (g) of the fresh ground NB meat (883.7 g) was higher than the gel strength of woody meat (720.8 g) with 0% phosphate (*p* ≤ 0.05). Addition of phosphate (0.25 and 0.5%) significantly increased the gel strength of fresh woody meat but it was significantly lower than NB meat added with 0.25 and 0.5% phosphate treatment. After freezing, ground NB meat samples with 0.25 and 0.5% phosphate had higher gel strength compared to fresh and frozen ground WB meat (*p* ≤ 0.05). Pre-blended STPP raised the pH in all treatments (*p* < 0.05). Treatments did not have any clear impact on APC of ground WB or NB meat. Addition of pre-blended sodium tripolyphosphate increases the functionality of fresh and frozen ground WB meat, as well as NB meat.

## 1. Introduction

In the United States, more chicken is consumed per capita than other protein choices, and the demand for poultry has been steadily growing over the past 50 years. More recently, chicken has dominated the world market as the most popular meat protein choice [1,2]. To keep up with consumer demand, poultry companies have increased production, improved breast yield, increased the growth rate, and extended grow-out times to produce more poultry meat; however, with these production improvements have come some meat quality issues such as woody breast (WB) [3]. In the past 5–10 years, WB has become a global concern for the world poultry industry It can be characterized by an abnormal hardness when the fillets are palpated and generally is paler in color than the normal breast (NB) fillets [4,5] and the WB meat is related to increased age and heavier grow-out in birds [6]. On a histological level, WB myofibrils have degeneration with necrosis and accumulating fibrosis (interstitial connective tissue) and lipidosis [7,8]. From a biochemical perspective, WB fillets are found to be higher in fat, moisture, and collagen, and lower in protein and ash [9,10]. The physiological and biochemical changes in the meat lead to poor texture, lower water holding capacity (WHC), higher drip loss, and poor eating quality. While there is no known way to control the formation of the woody breast meat during grow-out [11,12] the industry must find ways to add value to WB meat to reduce the economic losses.

Many processors try to remove the WB fillets from the processing line to redirect to further processed products. Previous research showed that by grinding the WB meat and cooking it as patties, they were able to improve the undesirable sensory characteristics of WB meat [13]. Ground WB meat can be used fresh or frozen for further processing. While this is a good way to utilize the poorer quality meat, there are some challenges in regards to high drip-loss, lower WHC, and higher cook loss [11,14,15] that accompany the use of WB meat in further processed products. While it is common practice within the industry to freeze ground meat in blocks to incorporate into further processed products, WB meat’s reduced quality can be further exacerbated by freezing, due to structural damage in the sarcolemma, myofibrils, and protein denaturation [16,17], which can be attributed to ice crystal formation, dehydration, and concentration of solutes [18]. Mitigating these quality changes will be important to protein functionality in further processed products.

Phosphates are used in meat processing for their impact on water binding, reduced oxidation, shelf-life extension, antimicrobial properties, and buffering capacity. They are also widely used in the production of surimi for their cryoprotective properties to preserve proteins by minimizing freezing damage [19]. Myosin and other muscle proteins that are important to functionality can be damaged in the freezing process; however, STPP can hydrate proteins by binding to the proteins and water causing decreased protein aggregation [20]. Some studies have suggested that phosphates may have the same cryoprotective ability to preserve protein functionality when used in poultry products [21,22,23,24]. However, many of these studies are focused on the use of washed poultry protein minces in surimi type products and combine the effects of multiple cryoprotectants. Functional ingredients can be added to aid in the binding of meat pieces, to improve water retention during all steps of processing, and to optimize finished product texture [25]. Adding sodium tripolyphosphate (STPP) as a pre-blend to coarsely ground meat that is intended to be used in further processing can possibly maintain quality properties of NB meat and help to improve the quality of WB meat. Coarsely ground meat can be defined as meat that still has recognizable meat fiber structures and would be more indicative of the type of meat that would be incorporated into further processed products.

Until the mechanism of WB is understood and the presence of this meat in the production is decreased, we must continue to understand the best ways to incorporate WB meat into further processed products to decrease economic losses. The current study is designed to determine the effect of pre-blending phosphate with the fresh ground WB meat compared to NB meat to determine if it will have any effect on the functional properties and the effect of pre-blending phosphate as a cryoprotectant during frozen storage of ground NB and WB meat.

## 2. Materials and Methods

In each of two replications, NB (68 kg) and WB (68 kg) fillets from approximately 8-week old birds were collected from a large-scale commercial processor in February and March of 2019 and transported to the laboratory at 4 °C. Each replication was collected on separate processing days. Fillets were scored, using hand palpation [15] so that only the most severe WB and NB fillets were used for further evaluation. Six treatments consisted of NB or WB meat with the addition of 0, 0.25, or 0.5% STPP (Brifsol 512, ICL Food Specialties, St. Louis, MO, USA) to each meat type and each of the six treatments were analyzed fresh and after a frozen storage period for 12 total treatments. Meat was divided into 22.7 kg batches (three batches of WB meat and three batches of NB meat) and each treatment was ground with a breaker plate (4.8 mm, MC32-18-187, Biro Manufacturing, Marblehead, OH, USA) in a mixer/grinder (Model Mini-32, BIRO Manufacturing, Marblehead, OH, USA) and then STPP was blended in the appropriate treatments and meat was ground with a finer plate (3.2 mm, MC32-18-125, Biro Manufacturing). After the final grinding step, each treatment was packaged separately (*n* = 10 samples/treatment × 12 treatments × 2 replications = 240), 1 kg of meat was added to a vacuum package bag (UltraSource, Kansas City, MO, USA) and half the bags were placed in refrigerated storage (4 °C), and half the bags were placed in a single layer and stored frozen (−20 °C) after the bags were sealed. Frozen (−20 °C) samples were stored for 6 d and moved from the freezer (−20 °C) to the refrigerator (4 °C) and thawed for 18 h prior to analysis.

All refrigerated and frozen ground meat samples were analyzed for pH, aerobic bacteria counts (APC), proximate analysis, color (CIE L*a*b*), and gel formation. pH was measured with a calibrated portable pH meter (HACH Company, Loveland, CO, USA) equipped with a spear tip probe (ISFET pH Stainless Steel Micro Probe Piercing Probe, HACH Company, Loveland, CO, USA).

### 2.1. Microbiological Analysis

Samples (*n* = 10/treatment/replication) were randomly selected for microbial analysis in each replication. From each sample, 25 g of meat were taken and placed into filter bags where 50 mL of 1% phosphate buffered saline (Fisher Scientific, Fair Lawn, NJ, USA) was added. All samples were homogenized in a stomacher for 60 s at 230 rpm. After homogenized, serial dilutions were prepared and plated in duplicate on standard methods agar (Acumedia Manufacturers Inc., Baltimore, MD, USA) and the plates were incubated at 37 °C for 24 h. Colonies were counted and reported as log cfu/g of meat.

### 2.2. Proximate Analysis

Ground meat samples (*n* = 10/treatment/replication) were randomly selected from each ground meat type, STPP level, and samples storage treatment for each replication. Samples were again homogenized using a small food chopper (Ninja Express Chop NJ100, SharkNinja Operating LLC, Needham, MA, USA). FOSS FoodScan™ with ISIscan™ software was used to determine the moisture, protein, and fat content of each sample. Once homogenized, a sample cup (D:140 mm, 14 mm height; FOSS Analytical A/S, Foss Allé 1, DK-3400, Hillerǿd, Denmark) was filled completely with sample and pressed into the plate. Each sample weighed approximately 250 g. Data were exported and duplicate runs were averaged for each sample for all parameters analyzed.

### 2.3. Color Analysis

Samples (*n* = 10) from each treatment and replication were subjected to color analysis. All meat was measured at 4 °C with a Minolta colorimeter (model CR-300/DP301, Minolta Corporation, Ramsey, NJ, USA) to determine the color utilizing the CIE L*a*b* color space. The equipment was calibrated with the white calibration plate, with a diffuse illuminant (D65) and 0° viewing geometry, and had an 8 mm measurement area. Three measurements in random spots in the ground meat were made on the surface of each sample and values were averaged by sample for statistical analysis of CIE L*, a*, and b* values.

### 2.4. Gel Preparation and Analysis

All ground meat samples (*n* = 10/treatment) were analyzed for gel formation in each replication. Briefly, 200 g of the meat sample, and 2 g of iodized salt (1% of the meat) was added to the small food chopper (model NJ100, Ninja Express Chop, SharkNinja Operating LLC, Needham, MA, USA) and homogenized (1 min) to form a meat slurry. Immediately, duplicate samples (35 ml) of the meat slurry were added to 50 ml conical tubes. The conical tubes were centrifuged (Sorvall Biofuge Stratos, Kendro Laboratory Products, Newtown, CT, USA) at 1000 rpm for 2 min at 4 °C to bring the meat slurry to the bottom of the tube and eliminate the air bubbles. The conical tubes were heated in a water bath (120 °C) until the meat slurry reached an internal temperature of 80 °C. The tubes were stored in a refrigerator (4 °C) overnight. The tubes were removed the following day and the meat was removed from the tube in a manner that did not disrupt the gel and any free water was drained off. All gels were analyzed for strength using a Texture Analyzer (model TA.XTplus, Texture Technologies Corp., Hamilton, MA/Stable Micro Systems, Godalming, Surrey, UK). The TA.XTplus was equipped with a 5 kg load cell and a ball-probe (TA-8). Gel strength was determined by the force in g it took to penetrate the cooked gel when the stainless-steel rod traveling at a constant rate of 100 mm/min.

### 2.5. Statistical Analysis

All microbial data was converted to log cfu/mL before analysis in the statistical model. Since zero cannot be statistically analyzed, a value of 0.69 log CFU/mL (lower detection limit) was assigned instead. Statistical Analysis of the data was conducted using SAS (SAS 9.4). Proc Glimmix Model of SAS was used to analyze the data to encompass the differences between two trials and comparisons were made using LSMEANS. Significant differences (*p* < 0.05) were identified.

## 3. Results and Discussion

While no solution to WB meat is available in the near future, we must continue to find ways to mitigate the financial losses associated with this less desirable meat product. One way to mitigate these losses is incorporate the WB meat into further processed products while finding solutions to the quality issues related to the WB meat. The solution investigated in this research is to incorporate STPP into ground meat as a pre-blend to help preserve the quality of fresh and frozen ground poultry meat. While STPP is already commonly used in many further processed products, adding STPP as a pre-blend to a course ground meat (fresh or frozen) to preserve functionality could add value to WB meat.

While phosphates are not considered to be a preservative, they can have bacteriostatic effects by slowing the growth of certain gram-positive bacteria [26]. In the current study, pre-blended STPP had no effect on aerobic plate counts (APCs) of ground NB or WB meat (Table 1). When all data was analyzed together, there were minor differences in APC counts. Overall, the APC counts were slightly higher (*p* < 0.05) in the frozen treatments; however, these treatments thawed for an 18 h time period at 4 °C before analysis which could allow for additional aerobic bacterial growth. There were no other clear trends in the APC data, and the largest differences were only 0.3 log cfu/g in difference (*p* < 0.05). Li et al. [27] found similar results when adding 0.5% STPP and distilled water to ground turkey meat, creating no differences in mesophilic bacterial counts compared to ground turkey treatments containing distilled water only. Since phosphates are considered to be bacteriostatic, it is possible that we would have more differences in data through a refrigerated storage study; however, with data points collected on the same day that the phosphate was added and just after thawing product, we only found small differences in the APCs in this study.

pH is an important measure in regard to protein functionality, WHC, and gelation properties. pH has also been shown to impact the aggregation and unfolding of proteins during heating, ultimately affecting the gel formation [28]. The ground WB meat has a higher pH (6.22) than the NB breast meat (6.15; Table 1). Soglia et al. [9] also reported that the ultimate pH of woody breast meat was higher than normal breast meat. Several researchers have reported higher pH values in WB fillets when measured approximately 4 h post mortem [13,29]. Contrarily, some researchers have reported no differences in pH between WB and NB meat [30,31]; however, some of these discrepancies in data could be attributed to methods of collection, sample size, or post-mortem age. Phosphates have the ability to buffer meat and move the pH away from the isoelectric point of the meat proteins. This distance between the isoelectric point and ultimate pH provides improvements in the water-holding properties of the proteins. All treatments that had phosphate addition had an increased (*p* < 0.05) pH when compared to no phosphate treatment (Table 1). Li et al. [27] found that the addition of STPP also increased the pH of ground turkey meat compared to ground meat with no STPP added. Freezing decreased (*p* < 0.05) the pH of NB and WB meat treatments, however, the treatments with added STPP had no change (*p* > 0.05) in pH compared to their corresponding fresh meat treatment (Table 1). Wei et al. [32] found decreasing pH values when poultry breast meat was subjected to frozen storage for 1 month or more, with values increasing as storage time increased. Nowsad et al. [21] also found a decrease in pH when spent hen mince was frozen. Freezing creates moisture loss in the meat, which can increase the concentration of solutes in the meat, which may explain the changes in the pH when the meat is thawed [18]. Additionally, the buffering capacity of the pre-blended STPP in the meat system did prevent a decrease in pH during frozen storage and actually raised the pH in some samples. pH values may have also been impacted by a change in WHC or the alkaline nature of the STPP.

Color was measured using the CIE L*a*b* color system, where L* values measure the lightness values with higher values equaling a lighter color, the a* value is a measure of redness with higher values indicating a more red color and the b* values equaling yellowness where higher values correlate to a more yellow color (Table 1). For the fresh ground WB and NB meat in this study, the WB meat was lighter in color than the ground NB meat (*p* < 0.05) with L* values of 62.9 and 57.9, respectively. While there are no other studies that look at the color of ground woody breast meat, studies have indicated that the color of woody breast meat is darker [33,34,35]; however, other studies have found no differences in color between the color of WB and NB meat [8,30]. Some researchers collect the color data on the ventral side of the fillet and some collect the data on the dorsal side of the fillet the difference in methods can lead to differences in the results [36]. However, in our study, where the color measurements were taken on ground WB and NB meat products, these types of inconsistencies would be eliminated. The addition of STPP as a pre-blend made all ground meat products lighter (*p* < 0.05) in color regardless of NB or WB category and all treatments were darker in color after frozen and thawed. The redness (a*) values were different among treatments (Table 1), with fresh ground WB meat having higher a* values (2.73) than fresh ground NB meat (2.16), indicating a more red product (*p* < 0.05) when subjected to instrumental measurement and the addition of STPP also elevated the a* values (*p* < 0.05). Frozen WB and NB had higher (*p* < 0.05) a* values than the fresh ground WB meat. This could be another indication of the cryoprotective properties of the STPP. The a* values in other studies looking at WB whole breast fillets have been higher in a* values than NB whole breast fillets [33,34,35] while other studies have found no differences [8,14]. The differences in redness values are very small, and while we found measurable differences by instrumental measurement, it is unlikely that differences would be visually noticeable. The b* values which indicate yellowness of a product followed a similar trend to that of the a* values. The fresh ground WB meat had higher (*p* < 0.05) b* values than fresh ground NB meat, indicating a more yellow product when subjected to instrumental measurement. In contrast to the trends in a* values, the addition of STPP lowered the b* values. Frozen WB and NB had higher (*p* < 0.05) b* values than the fresh ground WB meat and again, there were no differences (*p* > 0.05) between the yellowness values of the NB and WB with coinciding STPP treatments in the frozen ground meat treatments. As with the other color variables measured, there is conflicting data among researchers, which is likely due to sampling locations, sampling size or breed, and flock variations, on whole breast fillet but there is no data on the color of ground meat. Researchers have found WB whole fillets to be more yellow in color than NB fillets [6,14,33,34,35] while other researchers have found no differences in the yellowness values between WB and NB whole fillets [8,37]. Adding STPP as a pre-blend to ground WB and NB meat creates color changes; however, pre-blended meat is intended for use in further processed products and any color differences would likely go unnoticed when the ground meat is incorporated into a further processed product.

Proximate composition (Table 2) of samples can influence the functional properties and ultimately the protein gelation in a further processed product. Protein (%) in the fresh ground WB meat was lower (*p* < 0.05) than the fresh ground NB meat with values of 20.56 and 22.29%, respectively. This trend was consistent in treatments with STPP addition and samples subjected to frozen storage. Other researchers have also found a reduction in protein in WB meat [9,34,35]. Lower protein concentrations can have an impact on functionality and gel formation because this will provide fewer opportunities for protein–protein interaction in the gel. Samples with low protein content had elevated moisture and fat levels. Moisture was highest (*P* < 0.05) in fresh and frozen ground WB meat, 77.13 and 77.26%, respectively, when compared to all other treatments. Higher moisture in WB meat has also been reported by others [9,34,35]. The addition of STPP to all samples decreased moisture values, which could be due to the shift in water from free to immobilized phase affecting the NIR spectra. Fat (%) was also higher (*p* < 0.05) in ground WB meat than in the ground NB meat, and this has been reported throughout the literature [9,34,35]. During the growth of muscle, adipose tissue will replace damaged muscle cells, which will in turn slightly elevate fat percentages in WB meat. 

**Figure 1:**
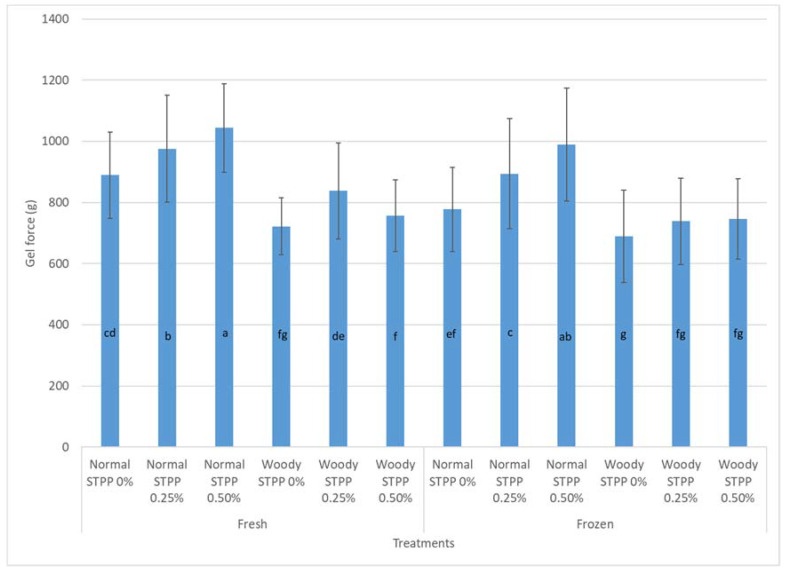
Gel strength (g) of fresh vs. frozen ground chicken breast meat prepared with woody and normal breast fillets with sodium tripolyphosphate (STPP; 0, 0.25, 0.5%) incorporated into the ground chicken meat (*n* = 20; Standard Error of Mean = 37.81). The dispersion bars represent standard deviation for each treatment. ^a–g^ Bars with common letters are not different (*p* > 0.05).

Protein gelation is one of the most important properties of protein functionality in further processed products and it can be largely impacted by frozen storage [24]. The gel strength of fresh ground NB and WB is significantly different, 883.7 g and 720.8 g, respectively (*p* < 0.05). Similarly, Xing et al. [37] also found that WB meat had lower gel strengths than NB meat. In the current study, pre-blending STPP improved gel strength in all samples except the fresh woody–0.5% STPP treatment where there was no improvement (Figure 1). In all other samples fresh and frozen, as levels of pre-blended STPP increased (*p* < 0.05) in the NB ground meat the gel strength increased. As expected, the frozen ground breast meat had lower gel strengths than the fresh ground breast meat treatments; however, the pre-blended STPP at 0.25 and 0.5% was able to preserve the protein functionality in the gel formation to levels similar to that of the frozen NB ground meat with no pre-blended STPP added. Additionally, the gel strength of the frozen ground WB meat with 0.25 and 0.50% STPP was similar (*p* > 0.05) to the fresh ground WB meat. This may also be an indication of STPPs cryoprotective ability in the WB meat. The gel strength of the frozen ground NB meat with no pre-blended STPP was no different (*p* > 0.05) from the fresh WB ground meat with no pre-blended STPP indicating some similarities in functionality in further processed products. STPP also raised (Table 1; *p* < 0.05) the pH of the meat system and higher pH in the meat system can improve gel formation by improving protein–protein interactions and WHC. Overall, using STPP as a pre-blend in ground fresh and frozen NB and WB meat helps improve protein functionality in terms of gel strength.

Chen et al. [30] used thawed NB and WB meat to prepare meatballs and found that there were vast differences in the microstructure of these cooked meatballs when they used microscopic methods to compare the samples. Xing et al. [37] also found difference in the microstructure of gels prepared with WB and NB meat, but were able to make some improvements in gel structure and texture by increasing the salt concentrations. These differences were likely because of the reduced protein content (Table 2) and partially denatured proteins of the WB meat. A reduction in salt soluble proteins or myofibrillar proteins can attribute to less protein-protein interactions during the formation of the gel matrix and these proteins are primarily responsible for texture in emulsified products, the reduction of these proteins can affect the gel matrix of a product [17,38]. Cai et al. [35] found that WB meat had less protein and salt soluble proteins than NB meat. Chen et al. [30] also stated that the microstructure of the gel formation of WB meat compared to NB meat has larger pores and aggregates that added to the low WHC of the meat. Freezing also creates protein denaturation and aggregates so this would likely compound the problem in utilizing frozen ground WB meat to form gels.

STPP can help protect proteins from cold denaturation, including myofibrillar proteins during frozen storage [18,24]. Frozen spent hen meat has improved gel properties when multiple cryoprotectants are used, including STPP; however, limited studies have been conducted to determine the cryoprotective ability of STPP in ground poultry meat [21,22,24]. Yoon et al. [19] was able to improve the water binding ability of whole chicken breasts and reduce the ice crystal formation during freezing when breast fillets were dipped in a 10% STPP solution, demonstrating the ability of STPP to function as a cryoprotectant on whole breast chicken meat. These researchers stated that the protection of the myofibrillar proteins and reduction of ice crystal formation were key factors in preserving the quality of the breast fillets. In the present study, the gel-strength of frozen NB without pre-blended STPP reduced significantly (*p* < 0.05) compared to the fresh NB without pre-blended STPP indicating lower functionality of the frozen meat. Addition of pre-blended STPP in NB demonstrated that the gel-strength of the frozen meat was higher (*p* < 0.05) than the 0% STPP and was similar (*p* > 0.05) to the fresh NB treatments. The data indicates that pre-blended STPP potentially demonstrated a cryoprotective effect on the meat by maintaining the functionality of the proteins after frozen storage. Gel-strength of fresh WB meat increased significantly after the addition of with 0.25% pre-blended STPP. However, the gel-strength of fresh 0.50% WB treatment was similar (*p* > 0.05) to the 0% WB meat. Further investigations must be conducted to determine the cryoprotective mechanism of STPP in NB and WB due to frozen storage.

## 4. Conclusions

Using a pre-blended STPP in NB and WB ground meat may provide cryoprotective benefits to the meat proteins, leading to better functionality and performance in further processed meat products. Furthermore, the functionality of WB with pre-blended STPP can be improved to levels similar to NB breast meat in some cases, in turn increasing the value of the ground WB meat. It is already a common practice to add ground chicken meat as a frozen ingredient into further processed products; therefore, pre-blending STPP into the ground product before addition to the further processed products could help improve functionality of NB and WB meat in products. It may also allow for incorporation of WB meat at higher inclusion rates to alleviate economic losses associated with WB meat while maintaining product quality in further processed products.

## Figures and Tables

**Table 1:** Color (CIE L*a*b*), pH, and aerobic plate count (APC) data for fresh vs. frozen ground chicken breast (normal and woody) treated with different levels of sodium tripolyphosphate (STPP; lsmean ± SD)^1^.

Ground Meat Storage	Treatment	L*	a*	b*	pH	APC (Log cfu/mL)
Fresh	Normal—0% STPP	57.93 ± 2.4 ^BC^	2.16 ± 0.48 ^F^	10.85 ± 2.10 ^EF^	6.15 ± 0.05 ^G^	3.06 ± 0.19 ^C^
	Normal—0.25% STPP	55.77 ± 2.6 ^DE^	1.98 ± 0.47 ^FG^	10.20 ± 1.87 ^FG^	6.28 ± 0.09 ^E^	3.11 ± 0.22 ^BC^
	Normal—0.50% STPP	53.19 ± 2.0 ^F^	1.66 ± 0.49 ^G^	9.30 ± 2.13 ^G^	6.33 ± 0.08 ^D^	3.06 ± 0.20 ^C^
	Woody—0% STPP	62.86 ± 2.4 ^A^	2.73 ± 0.62 ^DE^	12.19 ± 1.45 ^D^	6.22 ± 0.06 ^F^	3.20 ± 0.23 ^AB^
	Woody—0.25% STPP	59.01 ± 3.5 ^B^	2.35 ± 0.68 ^EF^	11.51 ± 1.90 ^DE^	6.38 ± 0.05 ^BC^	3.12 ± 0.20 ^BC^
	Woody—0.50% STPP	56.27 ± 3.4 ^D^	2.16 ± 0.32 ^F^	10.20 ± 2.5 ^FG^	6.40 ± 0.11 ^B^	3.34 ± 0.16 ^A^
Frozen	Normal—0% STPP	61.24 ± 2.8 ^A^	4.12 ± 1.09 ^A^	14.52 ± 3.03 ^AB^	6.11 ± 0.07 ^H^	3.23 ± 0.21 ^AB^
	Normal—0.25% STPP	57.35 ± 3.9 ^CD^	3.49 ± 1.11 ^B^	13.61 ± 3.04 ^BC^	6.34 ± 0.07 ^CD^	3.21 ± 0.18 ^AB^
	Normal—0.50% STPP	54.53 ± 3.0 ^EF^	2.87 ± 0.56 ^CD^	12.58 ± 2.30 ^C^	6.36 ± 0.07 ^CD^	3.24 ± 0.23 ^AB^
	Woody—0% STPP	61.25 ± 2.8 ^A^	4.20 ± 0.86 ^A^	15.36 ± 1.52 ^A^	6.20 ± 0.07 ^F^	3.16 ± 0.21 ^BC^
	Woody—0.25% STPP	59.06 ± 4.4 ^B^	3.75 ± 0.94 ^AB^	14.74 ± 2.75 ^AB^	6.37 ± 0.08 ^BC^	3.32 ± 0.26 ^A^
	Woody—0.50% STPP	58.17 ± 4.5 ^BC^	3.31 ± 1.03 ^BC^	13.37 ± 3.11 ^CD^	6.50 ± 0.09 ^A^	3.11 ± 0.24 ^BC^
	Standard Error of Mean	0.8502	0.2458	0.5969	0.0237	0.0899

^A–H^ Values with common superscripts within a column are not different (*p * > 0.05).^1^ *n* = 20.

**Table 2:** Proximate analysis (protein, moisture, and fat) data for fresh vs. frozen ground chicken breast (normal and woody) treated with different levels of sodium tripolyphosphate (STPP; lsmean ± SD).

Ground Meat Storage	Treatment ^1^	Protein (%)	Moisture (%)	Fat (%)
Fresh	Normal—0% STPP	22.29 ± 0.10 ^B^	76.19 ± 0.21 ^D^	2.14 ± 0.14 ^F^
	Normal—0.25% STPP	22.27 ± 0.13 ^B^	75.85 ± 0.17 ^E^	2.29 ± 0.13 ^DE^
	Normal—0.50% STPP	22.59 ± 0.30 ^A^	75.50 ± 0.42 ^F^	2.22 ± 0.23 ^EF^
	Woody—0% STPP	20.56 ± 0.10 ^F^	77.26 ± 0.29 ^A^	2.52 ± 0.20 ^C^
	Woody—0.25% STPP	21.04 ± 0.39 ^D^	76.66 ± 0.15 ^C^	2.60 ± 0.27 ^BC^
	Woody—0.50% STPP	20.88 ± 0.22 ^E^	76.87 ± 0.19 ^B^	2.64 ± 0.14 ^B^
Frozen	Normal—0% STPP	22.37 ± 0.21 ^B^	76.11 ± 0.26 ^D^	2.15 ± 0.09 ^F^
	Normal—0.25% STPP	22.16 ± 0.15^C^	75.84 ± 0.11 ^E^	2.37 ± 0.09 ^D^
	Normal—0.50% STPP	22.61 ± 0.38 ^A^	75.51 ± 0.32 ^F^	2.26 ± 0.11 ^E^
	Woody—0% STPP	20.50 ± 0.10 ^F^	77.13 ± 0.30 ^A^	2.53 ± 0.25 ^C^
	Woody—0.25% STPP	20.94 ± 0.28 ^E^	76.65 ± 0.19 ^C^	2.65 ± 0.22 ^AB^
	Woody—0.50% STPP	20.89 ± 0.25 ^E^	76.69 ± 0.13 ^C^	2.73 ± 0.15 ^A^
	Standard Error of Mean	0.1080	0.0963	0.0535

^A–F^ Values with common superscripts within a column are not different (*p* > 0.05). ^1^ *n * = 20.
